# The effect of library preparation protocol on the efficiency of heteroplasmy detection in mitochondrial DNA using two massively parallel sequencing Illumina systems

**DOI:** 10.1007/s13353-023-00821-4

**Published:** 2023-12-19

**Authors:** Patrycja Daca-Roszak, Joanna Fiedorowicz, Maciej Jankowski, Marzanna Ciesielka, Grzegorz Teresiński, Beata Lipska-Zietkiewicz, Ewa Zietkiewicz, Tomasz Grzybowski, Katarzyna Skonieczna

**Affiliations:** 1 Institute of Human Genetics, Polish Academy of Sciences, Poznan, Poland; 2 Present Address: Department of Animal Physiology, Biochemistry and Biostructure, Poznan University of Life Science, Poznan, Poland; 3 Department of Biology and Medical Genetics, Medical University of Gdansk, Gdansk, Poland; 4 Chair and Department of Forensic Medicine, Medical University of Lublin, Lublin, Poland; 5 Centre for Rare Diseases, Medical University of Gdansk, Gdansk, Poland; Clinical Genetics Unit, Department of Biology and Medical Genetics, Medical University of Gdansk, Gdansk, Poland; 6 Department of Forensic Medicine, Faculty of Medicine, Collegium Medicum, Nicolaus Copernicus University, Bydgoszcz, Poland

**Keywords:** mtDNA, Heteroplasmy detection, MPS library preparation, Nextera Flex, Nextera XT

## Abstract

Massively parallel sequencing (MPS) technology has become the gold standard in mitochondrial DNA research due to its high sensitivity in detecting mtDNA heteroplasmy, a prognostic marker in various medical applications. Various MPS technologies and platforms used for mtDNA analysis exist. Obtaining reliable and sensitive results requires deep and uniform coverage of the entire mtDNA sequence, which is heavily influenced by the choice of library preparation method and sequencing platform. Here, we present a comparison of the sequencing coverage and the ability to heteroplasmy detection using two library preparation protocols (*Nextera XT DNA Library Preparation Kit* and *Nextera DNA Flex Library Preparation Kit*) and two different (MiSeq FGx and ISeq 100) Illumina MPS platforms. Our study indicates that the Nextera DNA Flex Library protocol provides a more balanced coverage along the mitogenome and a reliable heteroplasmy detection with both MiSeq and iSeq Illumina MPS systems.

## Introduction

The mitochondrial DNA (mtDNA) genome is routinely analysed in many fields including forensic investigations, medical diagnostics, and comparative population studies (Payne et al. 2013; Brandhagen et al. 2020; McCormick et al. 2020).

One of the peculiarities of mtDNA, related to the high copy number of the mitogenome per cell, is the occurrence of heteroplasmy—the presence of major and minor variants at the same genomic position/s in a single individual (Wallace and Chalkia 2013). Heteroplasmy occurs not only preferentially in the non-coding D-loop encompassing hypervariable regions (HV1, HV2), but also throughout the whole mtDNA sequence (Stoneking 2000). While heteroplasmy may be a burden in phylogenetic or forensic studies, it is useful as a prognostic marker associated with age or mortality risk (Taylor and Turnbull 2005). Anyway, it is important to know what factors influence the detectable level of heteroplasmy in analysed samples.

Massively parallel sequencing (MPS) technology enables the detection of heteroplasmy at the level of 5% or even less (Taylor et al. 2020); however, obtaining reliable and sensitive results requires deep and uniform coverage of the entire mtDNA sequence, as well as application of proper bioinformatic tools for base calling quality assessment (Fazzini et al. 2021; Skonieczna and Grzybowski 2023). The choice of library preparation methodology and sequencing chemistry/platforms is also important, because some experimental conditions may promote the non-specific introduction of polymorphic variants into the sequence, resulting in false heteroplasmy signals (Fazzini et al. 2021). While the performance of various MPS technologies and platforms (e.g. Illumina, Ion Torrent, Roche 454-system) for mtDNA analysis has been extensively reported (Woerner et al. 2018; Fazzini et al. 2021), studies showing the importance of library preparation protocols for obtaining reliable MPS data are scarce (Obal et al. 2023).

Here, we present a comparison of sequencing coverage and mtDNA heteroplasmy detection using two library preparation protocols and two different Illumina MPS systems.

## Material and methods

DNA extracts from histopathologically normal colorectal samples of Polish colon cancer patients (*n* = 5; Skonieczna et al. 2018) were used as the source of mtDNA to compare the efficiency of various MPS protocols. Peripheral blood samples from healthy Polish individuals (*n* = 10) were used to confirm the performance of one of these protocols (see below). DNA was isolated using methods suitable for each tissue type: GeneMATRIX Bio-Trace DNA Purification Kit (EURX, Gdansk, Poland) for colorectal samples (Skonieczna et al. 2018) and Maxwell® RSC Blood DNA Kit for blood. Mitogenomes were amplified in two long-PCR reactions according to Fendt et al. (2009) protocol, using long PCR Enzyme Mix (Fermentas) and TaKaRa LA Taq DNA Polymerase (Takara Bio Inc., Kusatsu, Japan) for colon and blood samples, respectively.

Two different library preparation kits (both from Illumina, USA) were used, with the input DNA quantity ~ 200 ng: *Nextera XT DNA Library Preparation Kit (XT kit)* and *Nextera DNA Flex Library Preparation Kit (Flex kit)*. Protocols for preparing both types of libraries are similar, with the main difference in the tagmentation reaction (enzymatic DNA fragmentation and adapter ligation): in Nextera XT Kit it is carried out by transposomes in solution, whereas in Nextera Flex, it occurs on transposomes immobilized on beads.

Sequencing was performed using two Illumina systems generating paired-end reads: MiSeq FGx (2 × 300 bp) and iSeq 100 (2 × 150 bp). Colorectal samples were prepared and sequenced using three different protocols: (A) *XT* kit and MiSeq FGx; (B) *Flex* kit and MiSeq FGx; (C) *Flex* kit and iSeq 100. Blood samples (D) were processed with *Flex* kit and sequenced using iSeq 100. For colon samples, all XT libraries were sequenced in the same MiSeq FGx run, and all Flex libraries were sequenced in the same run on either MiSeq FGx or iSeq 100. All Flex libraries for blood samples were sequenced on iSeq 100 in five runs, independent from colon samples.

## Results and discussion

### The mean coverage and read depth

**Fig. 1:**
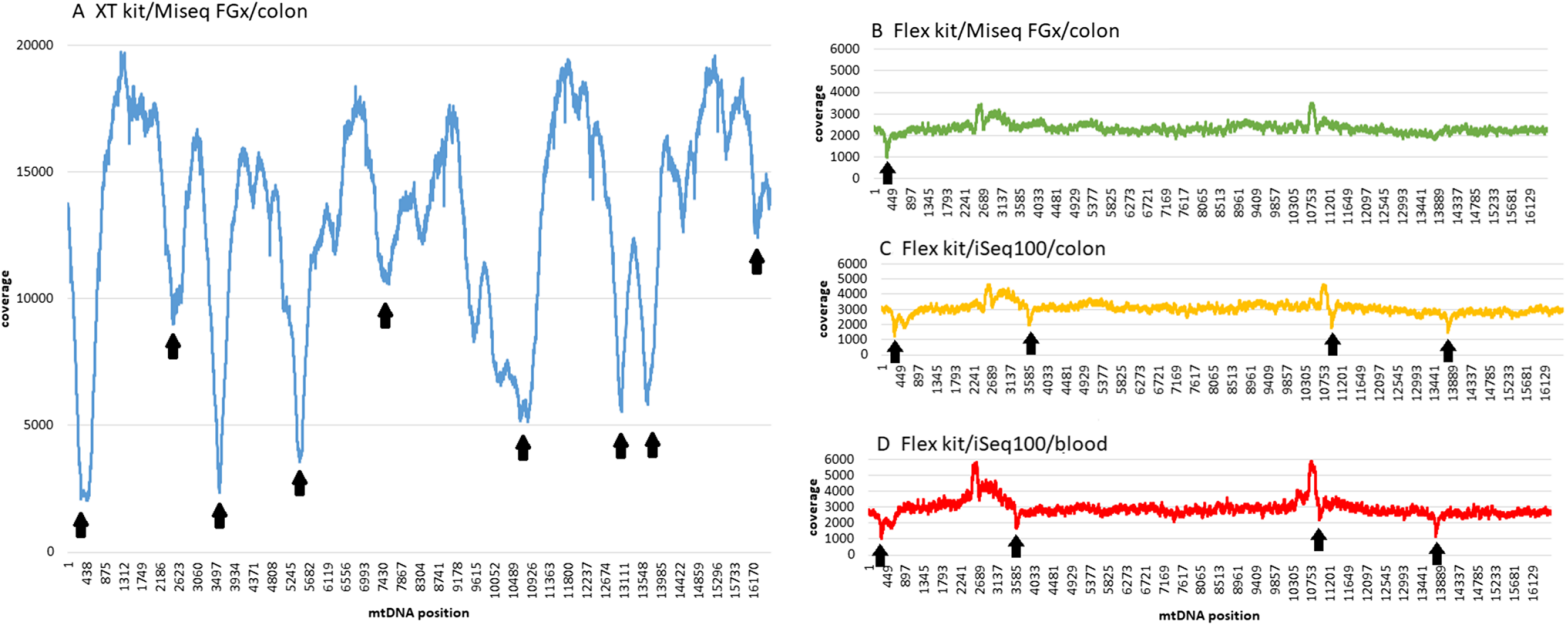
The mean coverage and the reads depth along the mtDNA sequence. All samples were subjected to the same bioinformatics analysis (Skonieczna and Grzybowski 2023). Homopolymer tracts between positions 303–315 and 16,180–16193 were excluded from the analysis. Arrows indicate regions with the decreased read depth. **A** In *Nextera XT DNA* libraries, several steep, haplotype-independent declines in the read depth were observed. **B**–**D** In *Nextera DNA Flex* libraries, the read depth was consistent along the whole mitogenome. In all *Nextera DNA Flex* libraries sequenced using iSeq100, a small but clear decrease in the coverage was observed in four regions (**C**, **D**—arrows). The decrease in the regions 290–501 and 13,608–13740 was associated with homopolymeric tracts in the mtDNA sequence, notorious for the difficulty of amplifying in PCR reaction at an early stage of sample preparation. The decrease in two other regions was the result of short reads (150 bp) generated by iSeq100, and the presence of several homopolymeric tracks (poly-C) between the nucleotides 3566–3589 and 11,427–11,524 (poly-C and poly-A), which hamper sequence alignment in shorts readings, but remain without a significant effect on longer reads (300 bp) generated from MiSeq. A slight increase in the coverage in two regions (2480–2688 and 10,658–10858) reflected the overlap of two PCR fragments (2 × 8.5 kbp) amplified in the initial step of *Nextera DNA Flex* library preparation

In both Illumina systems, MiSeq FGx and iSeq100, the mean coverage and the read depth along the genomic sequence strongly depended on the sequencing library preparation protocol (Fig. 1).

For *Nextera™ XT DNA* libraries, a high coverage was observed (mean 13,000 ×), but the read depth was very uneven along the mitogenome length (Fig. 1A). A similar observation has been described before and most likely reflected a sequence-specific bias of the Nextera transposase reaction and/or the library amplification of GC-rich regions (e.g. Ring et al. 2017).

*Nextera DNA Flex* libraries resulted in the lower level of mtDNA coverage (mean 2600 ×), but with the consistent read depth along the whole mitogenome (Fig. 1B–D). It is worth noting that the sequencing depth pattern was similar in both tissue types analysed (Fig. 1B–C versus D) and did not depend on the sequencing platform and polymerases used in PCR reaction (Fig. 1 B versus D). The coverage was only slightly higher when samples were sequenced using iSeq 100 as compared to MiSeq System (Fig. 1C, D versus B). In *Nextera DNA Flex* libraries sequenced using iseq100, a small decrease in the coverage was observed in four regions (see Fig. 1 caption for details).

### Heteroplasmy detection

Samples from histologically normal colon cells used to compare the efficiency of heteroplasmy detection using two library protocols and two different Illumina MPS platforms were selected to harbour heteroplasmic mutations (at least one) at the level of at least 2% (based on the previous results expected to be detectable by iSeq100 (Skonieczna and Grzybowski 2023)).

Sequencing of mitogenomes from the normal colon cells performed on Illumina platforms revealed heteroplasmy at the level of 3.38–38.48% (Table 1). Generally, similar results were obtained on the 454 Roche platform (5–41%) (Skonieczna et al. 2015). Similar to other studies, heteroplasmies occurring at the level of < 10% were undetectable using Sanger sequencing (e.g. McElhoe et al. 2022). Interestingly, some variants in the D-loop region (positions 16,362, 16,526, in samples 031 and 098), with the heteroplasmy at the level of 8% detected on 454 platform (in both normal and tumour tissue), were not seen using Illumina platforms. On the other hand, the presence of heteroplasmic mutations at these positions (16,362, 16,526) has been confirmed in the independent analysis performed in normal and altered tissue of the same patients (031 and 098—data not shown) and simultaneously excluded in 196 other samples sequenced on Illumina platforms (Skonieczna et al. 2018).

**Table 1:** Characteristics of the heteroplasmic variants detected in mitogenomes from 5 normal colon samples (the first five) and from 10 blood samples, using different library protocols and sequencing platforms

Sample	Position	Major variant	Minor variant frequency [%] and position’s coverage								
			XT/MiSeq FGx		Flex/MiSeq FGx			Flex/iSeq100		454 platform	Dideoxy
031	16,362	C	N	7823	N	2628	N		2204	T [8%]	N
033	114	T	C [19.27%]	6491	C [8%]	2660	C [8.04%]		3244	C [5%]	N
034	214	A	N	7583	G [3.38%]	2600	G [3.40%]		5022	G [5%]	N
	5935	A	G [29.48%]	15,911	G [26.38%]	2691	G [26.19%]		5432	G [30%]	conf
081	13,635	C	T [38.48%]	6969	T [32.86%]	931	T [37.23%]		1547	T [41%]	conf
098	16,526	A	N	28,230	N	1783	N		2022	G [8%]	N
1535	10,961	C						T [33.78]	2119		conf
1548	7165	C						T [44.31%]	1354		conf
1556	199	T						C [28.08%]	2108		conf

*XT/MiSeq FGx*: samples prepared with XT kit and sequenced on MiSeq FGx; *Flex/MiSeq FGx*: Flex kit and MiSeq FGx; *Flex/iSeq100*: Flex kit and iSeq 100; *454 platform*: GS FLX Titanium emPCR Kit (Lib-L)/454 system (Life Sciences); data published in Skonieczna et al. (2015). *Dideoxy –* resequenced with dideoxy sequencing; *N – *undetected; *conf –* heteroplasmy confirmed. In two colon samples (031 and 098), heteroplasmy at positions 16,362 and 16,526, detected only with 454 platform, was also found in the tumour tissue (Skonieczna and Grzybowski 2023). In seven blood samples (1211, 1219, 1565, 1572, 1573, 1580, 1584) analysed with Flex/ISeq100, no heteroplasmic positions were found. Validation of all detected heteroplasmic positions was carried out by dideoxy sequencing on 3130xl Genetic Analyser using BigDye™ Terminator v3.1 Cycle Sequencing Kit. In addition, the accuracy of the obtained sequences was confirmed by assigning haplotypes inferred from the informative positions to mtDNA haplogroups using Haplogrep 3 (Schönherr et al. 2023)

Such a lower (or inconsistent) sensitivity of Illumina platforms in detecting low-level variants from the D-loop has been reported earlier in comparison with the standard dideoxy method (Peck et al. 2016). Thus, it could not be ruled out that some stochastic variation at various steps of library preparation as well as the data reading technology itself may have influenced the level of minority variant in positions 16,362 and 16,526.

Although the 454 platform appears suitable for detecting heteroplasmic substitutions in the mitogenome (Holland et al. 2011; Suzuki et al. 2011), it is less cost-effective and more labour-consuming in comparison to Illumina technology. In consequence, it has been withdrawn from further production and largely replaced by various Illumina platform systems. We therefore focused here on the comparison between two Illumina systems, MiSeq and iSeq, in the context of different library preparation protocols.

Our study demonstrated that the reduced sensitivity of heteroplasmy detection in the D-loop region depended on the Illumina library preparation. For example, heteroplasmy at position 214 in sample 034 at the level of ≤ 5%, observed when using Flex Library protocol, was not detected using XT library protocol, despite the high level of coverage of this position. On the other hand, the XT library protocol suggested the level of 19% for the heteroplasmy at position 114 in sample 033, while Flex Library protocols indicated the heteroplasmy level of 8%. Both these examples corresponded to the non-uniform read depths observed in the mitogenome sequences prepared using the XT library, and pointed to a higher sensitivity and uniformity of heteroplasmy detection in samples prepared with Flex Library. Both MiSeq and iSeq systems when used in combination with the Flex libraries provided consistently similar detection of heteroplasmies.

Satisfactory parameters obtained in the sequencing of the colon tissue with a combination of Flex kit and iSeq100 System were confirmed using samples from the peripheral blood. In three out of 10 tested samples, heteroplasmy at the level of 28–44% (confirmed by dideoxy sequencing) was detected; no heteroplasmy at the lower level was observed. Importantly, the difference between the reported efficiency of heteroplasmy detection in normal colon tissue (3/5 samples) versus blood samples (3/10 samples) merely reflected the fact that the former had been preselected for harbouring at least one heteroplasmic position, rather than being due to tissue-specificity in the abundance of heteroplasmic positions. This was corroborated by the observation that the proportion of individuals with heteroplasmic substitutions detected using the Flex/iSeq protocol was comparable to that reported earlier in a larger number of normal colon tissues (Skonieczna et al. 2018). Simultaneously, caution should be taken regarding the results described here for blood samples, for which DNA isolation and amplification protocols differed from those used in the case of colon samples. Although detection of the minority variants above 28% was reliable (confirmed by the dideoxy method), the impact of different DNA preparation protocols on the sensitivity to detect lower frequency variants should be further examined. Furthermore, one has to be aware that due to the lack of technical replication, our blood results require validation on a larger study group.

## Conclusions

Both MiSeq and iSeq100 Illumina systems showed similar efficiency of heteroplasmy detection, as long as the libraries were prepared using the Flex kit. We therefore suggest that in low-budget and low-throughput laboratories, the combination of Nextera DNA Flex Library protocol with the less expensive iSeq100 Illumina platform provides a technically sound and cost-effective solution.

## Data Availability

Raw sequencing data are available under request.
