# Correction to: The effect of library preparation protocol on the efficiency of heteroplasmy detection in mitochondrial DNA using two massively parallel sequencing Illumina systems

**DOI:** 10.1007/s13353-024-00864-1

**Published:** 2024-04-13

**Authors:** Patrycja Daca‑Roszak, Joanna Fiedorowicz, Maciej Jankowski, Marzanna Ciesielka, Grzegorz Teresiński, Beata Lipska-Ziętkiewicz, Ewa Ziętkiewicz, Tomasz Grzybowski, Katarzyna Skonieczna

**Affiliations:** 1 Institute of Human Genetics, Polish Academy of Sciences, Poznan, Poland; 2 Present Address: Department of Animal Physiology, Biochemistry and Biostructure, Poznan University of Life Science, Poznan, Poland; 3 Department of Biology and Medical Genetics, Medical University of Gdansk, Gdansk, Poland; 4 Chair and Department of Forensic Medicine, Medical University of Lublin, Lublin, Poland; 5 Centre for Rare Diseases, Medical University of Gdansk, Gdansk, Poland; Clinical Genetics Unit, Department of Biology and Medical Genetics, Medical University of Gdansk, Gdansk, Poland; 6 Department of Forensic Medicine, Faculty of Medicine, Collegium Medicum, Nicolaus Copernicus University, Bydgoszcz, Poland

**Correction to: Journal of Applied Genetics (2024)**

10.1007/s13353-023-00821-4

The original article contains an error. The article was published with inverted names. Family name was captured first instead of the given names. This should be corrected as follows;

Patrycja Daca‑Roszak^1^ · Joanna Fiedorowicz^1,2^ · Maciej Jankowski^3^ · Marzanna Ciesielka^4^ ·Grzegorz Teresiński^4^ · Beata Lipska‑Zietkiewicz^5^ · Ewa Zietkiewicz^1^ · Tomasz Grzybowski^6^ · Katarzyna Skonieczna^6^

The original article has been corrected.

